# μ-Benzene-1,2,4,5-tetra­carboxyl­ato-κ^4^ *O* ^1^,*O* ^2^:*O* ^4^,*O* ^5^-bis­[diaqua(phen­an­thro­line-κ^2^ *N*,*N*′)nickel(II)] 0.67-hydrate

**DOI:** 10.1107/S1600536812004618

**Published:** 2012-02-10

**Authors:** Changfu Zhuang, Ning Li, Xiao-yang Yu

**Affiliations:** a Institute of Material Engineering, Southwest Forestry University, Kunming 650224, People’s Republic of China; b Pharmacy Department, Hospital of Faw, 2643 Dongfeng Street, Changchun, Jilin 130011, People’s Republic of China; c College of Chemical and Pharmaceutical Engineering, Jilin Institute of Chemical Technology, Jilin 132022, People’s Republic of China

## Abstract

The asymmetric unit of the title compound, [Ni_2_(C_10_H_2_O_8_)(C_12_H_8_N_2_)_2_(H_2_O)_4_]·0.67H_2_O, contains one complete binuclear complex and one half-mol­ecule, the latter being completed by crystallographic inversion symmetry, and 0.67 of a solvent water molecule. Each Ni^2+^ cation is coordinated by a 1,10-phenanthroline ligand, a bidentate benzene-1,2,4,5-tetra­carboxyl­ate (btec) tetra-anion and two water mol­ecules to generate a distorted *cis*-NiN_2_O_4_ octa­hedral coordination geometry. The btec species bridges the metal ions. In the crystal, the clusters and uncoordinated water mol­ecules are linked by O—H⋯O hydrogen bonds and π–π inter­actions [shortest centroid–centroid separation = 3.596 (2) Å] to form a three-dimensional network.

## Related literature

For btec as a ligand in coordination chemistry, see: Lu *et al.* (2005 ▶).
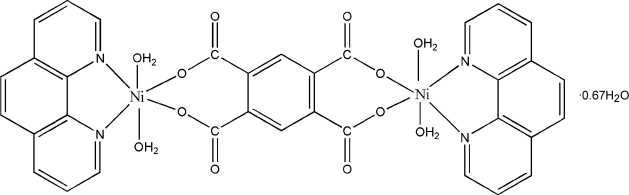


## Experimental

### 

#### Crystal data

[Ni_2_(C_10_H_2_O_8_)(C_12_H_8_N_2_)_2_(H_2_O)_4_]·0.67H_2_O
*M* *_r_* = 812.02
Triclinic, 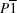

*a* = 9.855 (2) Å
*b* = 11.773 (2) Å
*c* = 21.803 (4) Å
α = 80.18 (3)°
β = 78.23 (3)°
γ = 75.27 (3)°
*V* = 2376.2 (8) Å^3^
*Z* = 3
Mo *K*α radiation
μ = 1.27 mm^−1^
*T* = 293 K
0.33 × 0.21 × 0.11 mm

#### Data collection

Bruker SMART CCD diffractometer
Absorption correction: multi-scan (*SADABS*; Sheldrick, 1996 ▶) *T*_min_ = 0.734, *T*_max_ = 0.870
20715 measured reflections
9302 independent reflections
6452 reflections with *I* > 2σ(*I*)
*R*_int_ = 0.038

#### Refinement

*R*[*F* ^2^ > 2σ(*F* ^2^)] = 0.049
*wR*(*F* ^2^) = 0.150
*S* = 1.03
9302 reflections
718 parameters
H atoms treated by a mixture of independent and constrained refinement
Δρ_max_ = 1.49 e Å^−3^
Δρ_min_ = −0.59 e Å^−3^

### 

Data collection: *SMART* (Bruker, 2001 ▶); cell refinement: *SAINT* (Bruker, 2001 ▶); data reduction: *SAINT*; program(s) used to solve structure: *SHELXS97* (Sheldrick, 2008 ▶); program(s) used to refine structure: *SHELXL97* (Sheldrick, 2008 ▶); molecular graphics: *SHELXTL* (Sheldrick, 2008 ▶); software used to prepare material for publication: *SHELXTL*.

## Supplementary Material

Crystal structure: contains datablock(s) I, global. DOI: 10.1107/S1600536812004618/hb6579sup1.cif

Structure factors: contains datablock(s) I. DOI: 10.1107/S1600536812004618/hb6579Isup2.hkl

Additional supplementary materials:  crystallographic information; 3D view; checkCIF report

## Figures and Tables

**Table 1:** Selected bond lengths (Å)

Ni1—O3	2.047 (3)
Ni1—O1*W*	2.073 (3)
Ni1—N8	2.074 (3)
Ni1—O2*W*	2.078 (3)
Ni1—N7	2.096 (3)
Ni1—O2	2.101 (3)
Ni2—O6	2.036 (3)
Ni2—O4*W*	2.054 (3)
Ni2—N10	2.079 (3)
Ni2—O3*W*	2.080 (3)
Ni2—N9	2.088 (3)
Ni2—O7	2.096 (3)
Ni3—O11	2.049 (3)
Ni3—O9	2.070 (3)
Ni3—O5*W*	2.079 (3)
Ni3—O6*W*	2.081 (3)
Ni3—N11	2.081 (3)
Ni3—N12	2.089 (3)

**Table 2:** Hydrogen-bond geometry (Å, °)

*D*—H⋯*A*	*D*—H	H⋯*A*	*D*⋯*A*	*D*—H⋯*A*
O1*W*—H1*WA*⋯O4^i^	0.81	1.97	2.774 (4)	176
O1*W*—H1*WB*⋯O8^ii^	0.88	1.81	2.667 (4)	163
O2*W*—H2*WA*⋯O7^iii^	0.82	2.32	3.122 (4)	168
O2*W*—H2*WB*⋯O3^i^	0.77	2.00	2.740 (4)	161
O3*W*—H3*WA*⋯O11	0.74	2.05	2.767 (4)	163
O3*W*—H3*WB*⋯O2^iv^	0.70	2.44	3.120 (4)	164
O4*W*—H4*WA*⋯O10^v^	0.89	1.87	2.746 (4)	166
O5*W*—H5*WA*⋯O6	0.78 (4)	1.92 (4)	2.692 (4)	173 (4)
O6*W*—H6*WA*⋯O5	0.76	2.12	2.874 (4)	174
O6*W*—H6*WB*⋯O1^iv^	0.87	1.80	2.622 (4)	156
O7*W*—H7*WA*⋯O9^vi^	0.85	2.05	2.893 (6)	172
O7*W*—H7*WB*⋯O12^vii^	0.85	2.15	2.797 (6)	132

Symmetry codes: (i) 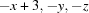; (ii) 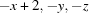; (iii) 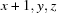; (iv) 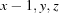; (v) 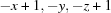; (vi) 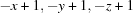; (vii) 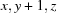.

**Fig. 1.**
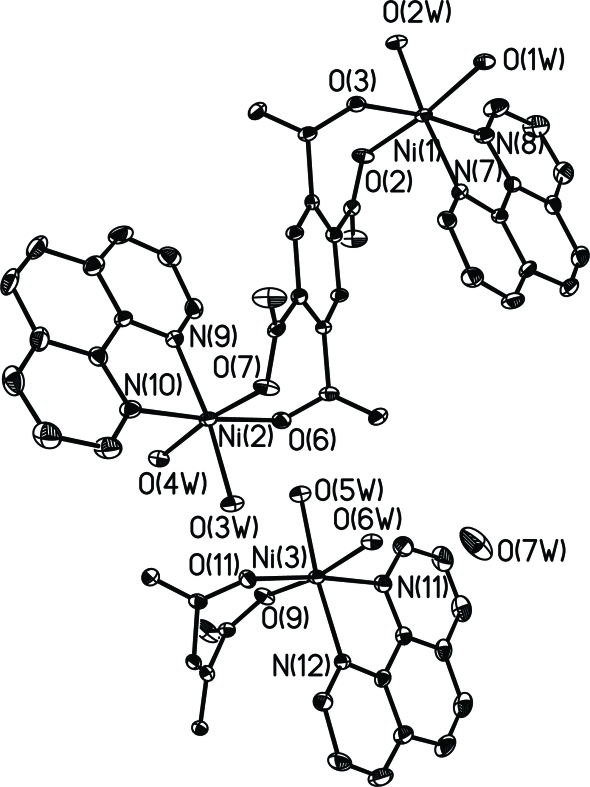
The asymmetric unit of the title compound with displacement ellipsoids drawn at the 30% probability level (H atoms omitted).

**Fig. 2.**
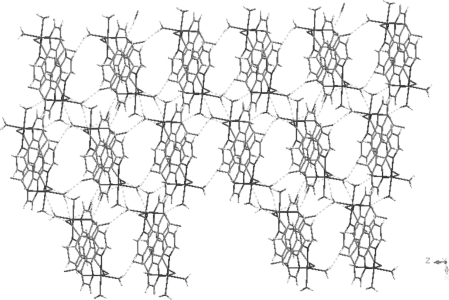
The hydrogen-bonding network of title compound, viewed down xz plane.

**Fig. 3.**
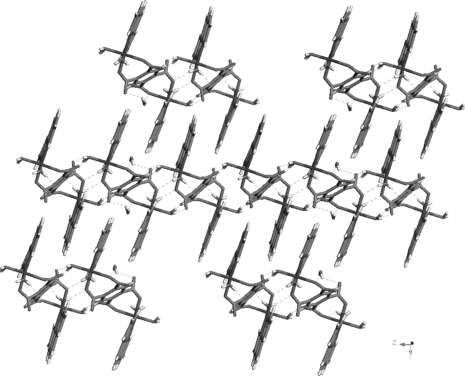
The π-π stacking network of title compound viewed down yz plane.

## supplementary crystallographic information

### Comment

As an organic ligand 1,2,4,5-benzenetetracarboxylic acid (H_4_btec) always attracts people's interest due to its rich coordination modes coordinating to metal ions through carboxyl groups(Lu *et al.*, 2005). Herein we are interested in compounds based on 1,2,4,5-benzenetetracarboxylic acid (H_4_btec) and 1,10-phenanthroline (phen) as ligands.To our knowledge, the coordination compounds based on these two ligands have been rare reported.

In the crystal structure of the title compound the Ni^2+^ cation is coordinated by two N atoms from phen, two O atoms of a btec and two O atoms of two coordinated water molecules, to form slightly distorted octahedra (Fig. 1). Two Ni^2+^ cations connect to each other through btec to form dinuclear cluster. These dinuclear clusters are connected *via* O—H···O hydrogen bonding into layers that are located in the *x*-*z*-plane (Fig. 2). Additional hydrogen bonds are also found between the H atoms of uncoordinated water and the O atoms of the btec. The π-π interaction occurs between phen molecules in the adjacent units resulting in a three-dimensional supramolecular network (Fig. 3).

### Experimental

NiSO_4_.6H_2_O (0.12 g), 1,10-phenanthroline (0.10 g), 1,2,4,5-benzenetetracarboxylic acid (0.07 g) and 18 ml water were mixed with stirring followed by adjusting the pH value to 5.0. Then the mixture was sealed in a 25 ml Teflon-lined stainless steel reactor and heated at 433 K for 72 h to give green blocks.

### Refinement

H atoms bonded to O atoms were located in a difference map and with *U*_iso_(H) = 1.2 (1.5 for uncoordinated water) times *U*_eq_(O). Other H atoms were positionedgeometrically and refined using a riding model, with C— H = 0.93 Å, and with *U*_iso_(H) = 1.2 *U*_eq_(C).

### Crystal data

**Table:**

[Ni_2_(C_10_H_2_O_8_)(C_12_H_8_N_2_)_2_(H_2_O)_4_]·0.67H_2_O	*Z* = 3
*M**_r_* = 812.02	*F*(000) = 1250
Triclinic, *P*1	*D*_x_ = 1.702 Mg m^−^^3^*D*_m_ = 1.702 Mg m^−^^3^*D*_m_ measured by not measured
*a* = 9.855 (2) Å	Mo *K*α radiation, λ = 0.71073 Å
*b* = 11.773 (2) Å	Cell parameters from 4291 reflections
*c* = 21.803 (4) Å	θ = 3.1–26°
α = 80.18 (3)°	µ = 1.27 mm^−^^1^
β = 78.23 (3)°	*T* = 293 K
γ = 75.27 (3)°	Block, green
*V* = 2376.2 (8) Å^3^	0.33 × 0.21 × 0.11 mm

### Data collection

**Table:**

Bruker SMART CCD diffractometer	9302 independent reflections
Radiation source: fine-focus sealed tube	6452 reflections with *I* > 2σ(*I*)
Graphite monochromator	*R*_int_ = 0.038
φ and ω scans	θ_max_ = 26.0°, θ_min_ = 3.1°
Absorption correction: multi-scan (*SADABS*; Sheldrick, 1996)	*h* = −12→12
*T*_min_ = 0.734, *T*_max_ = 0.870	*k* = −13→14
20715 measured reflections	*l* = −26→26

### Refinement

**Table:**

Refinement on *F*^2^	Primary atom site location: structure-invariant direct methods
Least-squares matrix: full	Secondary atom site location: difference Fourier map
*R*[*F*^2^ > 2σ(*F*^2^)] = 0.049	Hydrogen site location: inferred from neighbouring sites
*wR*(*F*^2^) = 0.150	H atoms treated by a mixture of independent and constrained refinement
*S* = 1.03	*w* = 1/[σ^2^(*F*_o_^2^) + (0.0772*P*)^2^ + 2.063*P*] where *P* = (*F*_o_^2^ + 2*F*_c_^2^)/3
9302 reflections	(Δ/σ)_max_ < 0.001
718 parameters	Δρ_max_ = 1.49 e Å^−^^3^
0 restraints	Δρ_min_ = −0.59 e Å^−^^3^

### Special details

**Table:**

Geometry. All e.s.d.'s (except the e.s.d. in the dihedral angle between two l.s. planes) are estimated using the full covariance matrix. The cell e.s.d.'s are taken into account individually in the estimation of e.s.d.'s in distances, angles and torsion angles; correlations between e.s.d.'s in cell parameters are only used when they are defined by crystal symmetry. An approximate (isotropic) treatment of cell e.s.d.'s is used for estimating e.s.d.'s involving l.s. planes.
Refinement. Refinement of *F*^2^ against ALL reflections. The weighted *R*-factor *wR* and goodness of fit *S* are based on *F*^2^, conventional *R*-factors *R* are based on *F*, with *F* set to zero for negative *F*^2^. The threshold expression of *F*^2^ > σ(*F*^2^) is used only for calculating *R*-factors(gt) *etc*. and is not relevant to the choice of reflections for refinement. *R*-factors based on *F*^2^ are statistically about twice as large as those based on *F*, and *R*- factors based on ALL data will be even larger.

### Fractional atomic coordinates and isotropic or equivalent isotropic displacement parameters (Å^2^)

**Table:**

	*x*	*y*	*z*	*U*_iso_*/*U*_eq_	
Ni1	1.33658 (4)	0.17132 (4)	0.05601 (2)	0.02006 (13)	
Ni2	0.64607 (5)	−0.13629 (4)	0.26400 (2)	0.02272 (14)	
Ni3	0.30687 (5)	0.20160 (4)	0.37780 (2)	0.02184 (14)	
O1	1.1679 (3)	0.1637 (3)	0.23881 (15)	0.0436 (8)	
O2	1.3206 (3)	0.1137 (3)	0.15331 (13)	0.0295 (6)	
O3	1.3218 (3)	0.0055 (2)	0.04573 (13)	0.0272 (6)	
O4	1.3435 (3)	−0.1676 (2)	0.10672 (16)	0.0404 (8)	
O5	0.6308 (3)	0.2003 (2)	0.20899 (16)	0.0385 (7)	
O6	0.6584 (3)	0.0300 (2)	0.27292 (13)	0.0288 (6)	
O7	0.6568 (3)	−0.0808 (3)	0.16690 (13)	0.0313 (7)	
O8	0.8078 (3)	−0.1403 (3)	0.08329 (15)	0.0449 (9)	
O9	0.3019 (3)	0.1501 (3)	0.47372 (13)	0.0319 (7)	
O10	0.2532 (4)	0.0810 (3)	0.57455 (15)	0.0516 (10)	
O11	0.2981 (3)	0.0343 (2)	0.36690 (13)	0.0303 (7)	
O12	0.3269 (3)	−0.1519 (2)	0.41068 (15)	0.0381 (7)	
C1	1.0202 (4)	0.2080 (4)	0.04246 (19)	0.0278 (9)	
H1	1.0447	0.1332	0.0294	0.033*	
C2	0.8775 (4)	0.2694 (4)	0.0488 (2)	0.0369 (10)	
H2	0.8080	0.2345	0.0421	0.044*	
C3	0.8419 (4)	0.3816 (4)	0.0652 (2)	0.0401 (11)	
H3	0.7473	0.4233	0.0697	0.048*	
C4	0.9461 (4)	0.4338 (4)	0.0751 (2)	0.0316 (9)	
C5	1.0862 (4)	0.3642 (3)	0.06979 (18)	0.0242 (8)	
C6	0.9213 (5)	0.5535 (4)	0.0884 (2)	0.0420 (11)	
H6	0.8295	0.6007	0.0913	0.050*	
C7	1.0268 (5)	0.5992 (4)	0.0968 (2)	0.0410 (11)	
H7	1.0065	0.6769	0.1056	0.049*	
C8	1.1706 (5)	0.5299 (3)	0.0923 (2)	0.0326 (10)	
C9	1.1996 (4)	0.4117 (3)	0.07934 (18)	0.0254 (8)	
C10	1.2861 (5)	0.5731 (4)	0.0989 (2)	0.0434 (12)	
H10	1.2726	0.6508	0.1067	0.052*	
C11	1.4181 (5)	0.5002 (5)	0.0940 (3)	0.0536 (15)	
H11	1.4953	0.5284	0.0982	0.064*	
C12	1.4386 (5)	0.3823 (4)	0.0826 (3)	0.0455 (13)	
H12	1.5293	0.3331	0.0808	0.055*	
C13	1.2023 (4)	0.1182 (3)	0.18932 (18)	0.0227 (8)	
C14	1.0949 (4)	0.0627 (3)	0.17247 (17)	0.0211 (8)	
C15	1.1320 (4)	−0.0222 (3)	0.13033 (17)	0.0193 (7)	
C16	1.2796 (4)	−0.0647 (3)	0.09339 (19)	0.0230 (8)	
C17	1.0245 (4)	−0.0675 (3)	0.11822 (17)	0.0209 (8)	
H17	1.0482	−0.1237	0.0902	0.025*	
C18	0.8828 (4)	−0.0325 (3)	0.14619 (17)	0.0206 (8)	
C19	0.8447 (4)	0.0545 (3)	0.18717 (17)	0.0202 (8)	
C20	0.9520 (4)	0.0999 (3)	0.19969 (17)	0.0218 (8)	
H20	0.9280	0.1569	0.2272	0.026*	
C21	0.6974 (4)	0.0990 (3)	0.22358 (19)	0.0243 (8)	
C22	0.7757 (4)	−0.0904 (3)	0.13044 (19)	0.0227 (8)	
C23	0.9600 (4)	−0.1727 (4)	0.2815 (2)	0.0320 (9)	
H23	0.9357	−0.0956	0.2915	0.038*	
C24	1.1006 (5)	−0.2361 (4)	0.2803 (2)	0.0421 (11)	
H24	1.1692	−0.2007	0.2876	0.051*	
C25	1.1360 (5)	−0.3513 (5)	0.2683 (2)	0.0443 (12)	
H25	1.2296	−0.3943	0.2666	0.053*	
C26	1.0312 (5)	−0.4046 (4)	0.2586 (2)	0.0370 (10)	
C27	0.8938 (4)	−0.3329 (3)	0.25910 (19)	0.0265 (8)	
C28	1.0560 (5)	−0.5263 (4)	0.2492 (2)	0.0448 (12)	
H28	1.1472	−0.5743	0.2483	0.054*	
C29	0.9500 (6)	−0.5721 (4)	0.2416 (2)	0.0443 (12)	
H29	0.9689	−0.6517	0.2363	0.053*	
C30	0.8080 (5)	−0.5015 (4)	0.2414 (2)	0.0380 (11)	
C31	0.7807 (4)	−0.3810 (4)	0.24947 (19)	0.0282 (9)	
C32	0.6931 (6)	−0.5433 (4)	0.2331 (2)	0.0493 (13)	
H32	0.7055	−0.6227	0.2287	0.059*	
C33	0.5638 (6)	−0.4690 (5)	0.2312 (3)	0.0525 (14)	
H33	0.4881	−0.4965	0.2247	0.063*	
C34	0.5447 (5)	−0.3485 (4)	0.2395 (2)	0.0454 (13)	
H34	0.4557	−0.2977	0.2382	0.055*	
C35	0.3967 (5)	0.4274 (4)	0.3927 (2)	0.0428 (12)	
H35	0.4907	0.3861	0.3829	0.051*	
C36	0.3697 (6)	0.5441 (4)	0.4076 (3)	0.0533 (14)	
H36	0.4449	0.5782	0.4079	0.064*	
C37	0.2341 (6)	0.6059 (4)	0.4214 (2)	0.0497 (13)	
H37	0.2155	0.6824	0.4318	0.060*	
C38	0.1206 (5)	0.5541 (4)	0.4199 (2)	0.0366 (10)	
C39	0.1570 (4)	0.4380 (3)	0.40465 (19)	0.0264 (8)	
C40	−0.0256 (6)	0.6138 (4)	0.4319 (2)	0.0481 (13)	
H40	−0.0494	0.6904	0.4426	0.058*	
C41	−0.1303 (5)	0.5618 (4)	0.4280 (3)	0.0512 (13)	
H41	−0.2246	0.6034	0.4355	0.061*	
C42	−0.0977 (4)	0.4429 (4)	0.4126 (2)	0.0364 (10)	
C43	0.0463 (4)	0.3822 (3)	0.40086 (18)	0.0272 (9)	
C44	−0.1991 (5)	0.3830 (5)	0.4076 (2)	0.0487 (13)	
H44	−0.2955	0.4184	0.4168	0.058*	
C45	−0.1568 (5)	0.2716 (5)	0.3891 (2)	0.0438 (12)	
H45	−0.2240	0.2311	0.3856	0.053*	
C46	−0.0119 (4)	0.2202 (4)	0.3757 (2)	0.0312 (9)	
H46	0.0160	0.1471	0.3606	0.037*	
C47	0.2329 (4)	0.0947 (3)	0.52013 (19)	0.0250 (8)	
C48	0.1144 (4)	0.0455 (3)	0.50735 (17)	0.0222 (8)	
C49	−0.0140 (4)	0.0623 (3)	0.54958 (17)	0.0215 (8)	
H49	−0.0240	0.1045	0.5832	0.026*	
C50	0.1278 (4)	−0.0179 (3)	0.45705 (17)	0.0222 (8)	
C51	0.2623 (4)	−0.0463 (3)	0.40893 (18)	0.0240 (8)	
N7	1.1215 (3)	0.2523 (3)	0.05425 (15)	0.0231 (7)	
N8	1.3321 (3)	0.3394 (3)	0.07449 (17)	0.0293 (8)	
N9	0.8590 (3)	−0.2172 (3)	0.26891 (15)	0.0262 (7)	
N10	0.6501 (3)	−0.3068 (3)	0.24880 (17)	0.0299 (8)	
N11	0.2936 (3)	0.3747 (3)	0.39206 (16)	0.0292 (8)	
N12	0.0879 (3)	0.2706 (3)	0.38354 (15)	0.0235 (7)	
O1W	1.3809 (3)	0.2204 (2)	−0.04060 (12)	0.0269 (6)	
H1WA	1.4622	0.2074	−0.0587	0.032*	
H1WB	1.3328	0.1819	−0.0561	0.032*	
O2W	1.5531 (3)	0.1097 (2)	0.05774 (13)	0.0306 (6)	
H2WA	1.5714	0.0667	0.0902	0.037*	
H2WB	1.6005	0.0876	0.0277	0.037*	
O3W	0.4295 (3)	−0.0771 (2)	0.26125 (13)	0.0310 (6)	
H3WA	0.3860	−0.0584	0.2914	0.037*	
H3WB	0.4167	−0.0414	0.2328	0.037*	
O4W	0.6068 (3)	−0.1815 (2)	0.36013 (13)	0.0306 (6)	
H4WA	0.6427	−0.1377	0.3794	0.037*	
H4WB	0.5341	−0.1764	0.3784	0.037*	
O5W	0.5271 (3)	0.1520 (3)	0.37057 (16)	0.0329 (7)	
H5WA	0.559 (5)	0.114 (4)	0.343 (2)	0.039*	
H5WB	0.563 (5)	0.113 (4)	0.401 (2)	0.039*	
O6W	0.3478 (3)	0.2493 (2)	0.28060 (13)	0.0282 (6)	
H6WA	0.4223	0.2414	0.2612	0.034*	
H6WB	0.3107	0.2088	0.2612	0.034*	
O7W	0.4711 (6)	0.7469 (7)	0.5118 (3)	0.150 (3)	
H7WA	0.5412	0.7759	0.5125	0.225*	
H7WB	0.4057	0.8027	0.4990	0.225*	

### Atomic displacement parameters (Å^2^)

**Table:**

	*U*^11^	*U*^22^	*U*^33^	*U*^12^	*U*^13^	*U*^23^
Ni1	0.0133 (2)	0.0212 (3)	0.0262 (3)	−0.00299 (19)	−0.00116 (18)	−0.0085 (2)
Ni2	0.0161 (2)	0.0240 (3)	0.0280 (3)	−0.0033 (2)	−0.00058 (19)	−0.0087 (2)
Ni3	0.0176 (2)	0.0218 (3)	0.0268 (3)	−0.0049 (2)	−0.00064 (19)	−0.0081 (2)
O1	0.0295 (16)	0.066 (2)	0.044 (2)	−0.0147 (16)	−0.0015 (14)	−0.0321 (17)
O2	0.0169 (13)	0.0458 (18)	0.0280 (15)	−0.0098 (13)	−0.0018 (11)	−0.0092 (13)
O3	0.0243 (13)	0.0246 (14)	0.0316 (16)	−0.0061 (12)	0.0039 (12)	−0.0098 (12)
O4	0.0236 (15)	0.0226 (15)	0.065 (2)	0.0008 (13)	0.0049 (14)	−0.0028 (15)
O5	0.0262 (15)	0.0243 (15)	0.056 (2)	0.0014 (13)	0.0032 (14)	−0.0050 (14)
O6	0.0283 (14)	0.0271 (14)	0.0291 (15)	−0.0057 (12)	0.0050 (12)	−0.0108 (12)
O7	0.0193 (13)	0.0517 (19)	0.0265 (15)	−0.0138 (13)	0.0007 (11)	−0.0114 (13)
O8	0.0290 (16)	0.071 (2)	0.045 (2)	−0.0150 (16)	−0.0006 (14)	−0.0360 (18)
O9	0.0290 (15)	0.0439 (18)	0.0267 (15)	−0.0182 (14)	−0.0023 (12)	−0.0034 (13)
O10	0.056 (2)	0.086 (3)	0.0286 (18)	−0.047 (2)	−0.0155 (15)	0.0042 (17)
O11	0.0345 (15)	0.0202 (14)	0.0317 (16)	−0.0072 (12)	0.0099 (13)	−0.0084 (12)
O12	0.0295 (15)	0.0239 (15)	0.053 (2)	−0.0029 (13)	0.0065 (14)	−0.0038 (14)
C1	0.0220 (19)	0.032 (2)	0.030 (2)	−0.0089 (18)	−0.0059 (16)	0.0002 (17)
C2	0.024 (2)	0.045 (3)	0.042 (3)	−0.009 (2)	−0.0096 (19)	0.001 (2)
C3	0.019 (2)	0.049 (3)	0.043 (3)	0.004 (2)	−0.0020 (18)	−0.003 (2)
C4	0.026 (2)	0.030 (2)	0.030 (2)	0.0081 (18)	−0.0037 (17)	−0.0002 (18)
C5	0.0217 (18)	0.026 (2)	0.0194 (19)	0.0000 (16)	0.0011 (15)	−0.0019 (16)
C6	0.037 (2)	0.034 (2)	0.040 (3)	0.011 (2)	0.001 (2)	−0.001 (2)
C7	0.055 (3)	0.022 (2)	0.037 (3)	0.003 (2)	0.003 (2)	−0.0082 (19)
C8	0.046 (3)	0.0195 (19)	0.028 (2)	−0.0053 (19)	0.0037 (19)	−0.0062 (17)
C9	0.028 (2)	0.024 (2)	0.0202 (19)	−0.0024 (17)	0.0022 (16)	−0.0052 (16)
C10	0.059 (3)	0.023 (2)	0.051 (3)	−0.014 (2)	0.000 (2)	−0.014 (2)
C11	0.046 (3)	0.046 (3)	0.078 (4)	−0.023 (3)	0.000 (3)	−0.028 (3)
C12	0.029 (2)	0.042 (3)	0.070 (4)	−0.009 (2)	−0.003 (2)	−0.025 (3)
C13	0.0212 (18)	0.0231 (19)	0.026 (2)	−0.0053 (16)	−0.0066 (16)	−0.0056 (16)
C14	0.0193 (17)	0.0222 (19)	0.023 (2)	−0.0071 (16)	−0.0024 (15)	−0.0051 (15)
C15	0.0198 (17)	0.0167 (17)	0.0206 (19)	−0.0047 (15)	−0.0017 (14)	−0.0016 (14)
C16	0.0170 (17)	0.023 (2)	0.031 (2)	−0.0052 (16)	0.0003 (15)	−0.0119 (17)
C17	0.0179 (17)	0.0201 (18)	0.025 (2)	−0.0044 (15)	0.0014 (15)	−0.0088 (15)
C18	0.0189 (17)	0.0193 (18)	0.025 (2)	−0.0052 (15)	−0.0027 (15)	−0.0049 (15)
C19	0.0150 (16)	0.0204 (18)	0.0229 (19)	−0.0022 (15)	0.0002 (14)	−0.0036 (15)
C20	0.0189 (17)	0.0220 (19)	0.024 (2)	−0.0032 (16)	0.0018 (15)	−0.0103 (16)
C21	0.0169 (17)	0.026 (2)	0.032 (2)	−0.0062 (16)	0.0006 (16)	−0.0131 (17)
C22	0.0163 (17)	0.0204 (19)	0.031 (2)	−0.0004 (15)	−0.0063 (15)	−0.0050 (16)
C23	0.031 (2)	0.031 (2)	0.035 (2)	−0.0096 (19)	−0.0115 (18)	0.0030 (18)
C24	0.029 (2)	0.056 (3)	0.041 (3)	−0.012 (2)	−0.009 (2)	0.003 (2)
C25	0.022 (2)	0.055 (3)	0.045 (3)	0.009 (2)	−0.0081 (19)	−0.001 (2)
C26	0.036 (2)	0.038 (2)	0.027 (2)	0.005 (2)	−0.0031 (19)	0.0001 (19)
C27	0.0219 (19)	0.026 (2)	0.026 (2)	0.0003 (17)	0.0004 (16)	−0.0023 (16)
C28	0.049 (3)	0.032 (2)	0.039 (3)	0.013 (2)	−0.005 (2)	0.000 (2)
C29	0.064 (3)	0.019 (2)	0.040 (3)	0.006 (2)	−0.003 (2)	−0.0058 (19)
C30	0.052 (3)	0.025 (2)	0.032 (2)	−0.005 (2)	0.001 (2)	−0.0034 (18)
C31	0.031 (2)	0.028 (2)	0.025 (2)	−0.0090 (18)	−0.0010 (17)	−0.0027 (17)
C32	0.070 (4)	0.032 (3)	0.051 (3)	−0.021 (3)	−0.003 (3)	−0.011 (2)
C33	0.050 (3)	0.052 (3)	0.066 (4)	−0.027 (3)	−0.001 (3)	−0.024 (3)
C34	0.034 (2)	0.046 (3)	0.063 (3)	−0.015 (2)	0.002 (2)	−0.027 (3)
C35	0.037 (2)	0.037 (3)	0.060 (3)	−0.016 (2)	−0.012 (2)	−0.005 (2)
C36	0.066 (4)	0.037 (3)	0.071 (4)	−0.031 (3)	−0.015 (3)	−0.011 (3)
C37	0.075 (4)	0.029 (2)	0.052 (3)	−0.020 (3)	−0.009 (3)	−0.013 (2)
C38	0.051 (3)	0.025 (2)	0.032 (2)	−0.006 (2)	−0.004 (2)	−0.0053 (18)
C39	0.032 (2)	0.0231 (19)	0.025 (2)	−0.0066 (17)	−0.0042 (17)	−0.0056 (16)
C40	0.064 (3)	0.024 (2)	0.049 (3)	0.004 (2)	−0.003 (3)	−0.013 (2)
C41	0.046 (3)	0.034 (3)	0.057 (3)	0.014 (2)	0.002 (2)	−0.009 (2)
C42	0.030 (2)	0.035 (2)	0.039 (3)	0.000 (2)	−0.0022 (19)	−0.004 (2)
C43	0.029 (2)	0.023 (2)	0.025 (2)	0.0001 (17)	−0.0017 (16)	−0.0045 (16)
C44	0.026 (2)	0.056 (3)	0.056 (3)	0.002 (2)	−0.004 (2)	−0.006 (3)
C45	0.027 (2)	0.054 (3)	0.055 (3)	−0.020 (2)	−0.011 (2)	0.002 (2)
C46	0.029 (2)	0.032 (2)	0.034 (2)	−0.0085 (19)	−0.0066 (18)	−0.0040 (18)
C47	0.0202 (18)	0.028 (2)	0.027 (2)	−0.0057 (17)	−0.0032 (16)	−0.0052 (17)
C48	0.0234 (18)	0.0213 (19)	0.023 (2)	−0.0078 (16)	−0.0036 (15)	−0.0018 (15)
C49	0.0208 (18)	0.0225 (19)	0.0217 (19)	−0.0057 (16)	0.0007 (15)	−0.0083 (15)
C50	0.0214 (18)	0.0212 (19)	0.023 (2)	−0.0069 (16)	0.0013 (15)	−0.0030 (15)
C51	0.0195 (18)	0.028 (2)	0.027 (2)	−0.0086 (17)	−0.0029 (15)	−0.0071 (17)
N7	0.0220 (16)	0.0198 (16)	0.0254 (17)	−0.0019 (13)	−0.0017 (13)	−0.0039 (13)
N8	0.0227 (16)	0.0293 (18)	0.038 (2)	−0.0082 (15)	−0.0001 (15)	−0.0131 (16)
N9	0.0225 (16)	0.0257 (17)	0.0286 (18)	−0.0034 (14)	−0.0042 (14)	−0.0024 (14)
N10	0.0248 (17)	0.0297 (18)	0.036 (2)	−0.0060 (15)	0.0012 (15)	−0.0137 (15)
N11	0.0299 (18)	0.0257 (17)	0.034 (2)	−0.0100 (15)	−0.0027 (15)	−0.0089 (15)
N12	0.0206 (15)	0.0227 (16)	0.0264 (17)	−0.0046 (14)	−0.0023 (13)	−0.0037 (13)
O1W	0.0167 (12)	0.0355 (16)	0.0301 (15)	−0.0063 (12)	−0.0027 (11)	−0.0096 (12)
O2W	0.0186 (13)	0.0387 (16)	0.0320 (16)	0.0004 (12)	−0.0012 (11)	−0.0118 (13)
O3W	0.0198 (13)	0.0401 (17)	0.0300 (16)	−0.0006 (13)	−0.0008 (11)	−0.0094 (13)
O4W	0.0235 (14)	0.0367 (16)	0.0313 (16)	−0.0084 (13)	−0.0004 (12)	−0.0065 (13)
O5W	0.0240 (15)	0.0396 (18)	0.0339 (18)	−0.0031 (14)	−0.0034 (13)	−0.0102 (14)
O6W	0.0209 (13)	0.0361 (16)	0.0293 (15)	−0.0069 (12)	−0.0022 (11)	−0.0104 (12)
O7W	0.122 (5)	0.256 (8)	0.096 (4)	−0.121 (6)	−0.064 (4)	0.086 (5)

### Geometric parameters (Å, º)

**Table:**

Ni1—O3	2.047 (3)	C23—H23	0.9300
Ni1—O1W	2.073 (3)	C24—C25	1.369 (7)
Ni1—N8	2.074 (3)	C24—H24	0.9300
Ni1—O2W	2.078 (3)	C25—C26	1.404 (6)
Ni1—N7	2.096 (3)	C25—H25	0.9300
Ni1—O2	2.101 (3)	C26—C27	1.401 (6)
Ni2—O6	2.036 (3)	C26—C28	1.434 (6)
Ni2—O4W	2.054 (3)	C27—N9	1.361 (5)
Ni2—N10	2.079 (3)	C27—C31	1.437 (5)
Ni2—O3W	2.080 (3)	C28—C29	1.341 (7)
Ni2—N9	2.088 (3)	C28—H28	0.9300
Ni2—O7	2.096 (3)	C29—C30	1.434 (7)
Ni3—O11	2.049 (3)	C29—H29	0.9300
Ni3—O9	2.070 (3)	C30—C32	1.399 (7)
Ni3—O5W	2.079 (3)	C30—C31	1.409 (6)
Ni3—O6W	2.081 (3)	C31—N10	1.361 (5)
Ni3—N11	2.081 (3)	C32—C33	1.353 (8)
Ni3—N12	2.089 (3)	C32—H32	0.9300
O1—C13	1.234 (5)	C33—C34	1.420 (6)
O2—C13	1.260 (4)	C33—H33	0.9300
O3—C16	1.281 (5)	C34—N10	1.319 (5)
O4—C16	1.231 (5)	C34—H34	0.9300
O5—C21	1.229 (5)	C35—N11	1.322 (5)
O6—C21	1.287 (5)	C35—C36	1.412 (6)
O7—C22	1.265 (4)	C35—H35	0.9300
O8—C22	1.217 (5)	C36—C37	1.350 (7)
O9—C47	1.277 (5)	C36—H36	0.9300
O10—C47	1.220 (5)	C37—C38	1.412 (6)
O11—C51	1.267 (5)	C37—H37	0.9300
O12—C51	1.243 (5)	C38—C39	1.402 (6)
C1—N7	1.323 (5)	C38—C40	1.425 (7)
C1—C2	1.399 (6)	C39—N11	1.361 (5)
C1—H1	0.9300	C39—C43	1.432 (5)
C2—C3	1.367 (6)	C40—C41	1.349 (7)
C2—H2	0.9300	C40—H40	0.9300
C3—C4	1.392 (6)	C41—C42	1.436 (6)
C3—H3	0.9300	C41—H41	0.9300
C4—C5	1.408 (5)	C42—C44	1.391 (6)
C4—C6	1.437 (6)	C42—C43	1.410 (6)
C5—N7	1.358 (5)	C43—N12	1.367 (5)
C5—C9	1.434 (5)	C44—C45	1.376 (7)
C6—C7	1.340 (7)	C44—H44	0.9300
C6—H6	0.9300	C45—C46	1.394 (6)
C7—C8	1.438 (6)	C45—H45	0.9300
C7—H7	0.9300	C46—N12	1.322 (5)
C8—C10	1.401 (6)	C46—H46	0.9300
C8—C9	1.413 (5)	C47—C48	1.520 (5)
C9—N8	1.360 (5)	C48—C50	1.395 (5)
C10—C11	1.359 (7)	C48—C49	1.396 (5)
C10—H10	0.9300	C49—C50^i^	1.393 (5)
C11—C12	1.408 (6)	C49—H49	0.9300
C11—H11	0.9300	C50—C49^i^	1.393 (5)
C12—N8	1.328 (5)	C50—C51	1.515 (5)
C12—H12	0.9300	O1W—H1WA	0.8064
C13—C14	1.507 (5)	O1W—H1WB	0.8787
C14—C20	1.400 (5)	O2W—H2WA	0.8203
C14—C15	1.402 (5)	O2W—H2WB	0.7666
C15—C17	1.388 (5)	O3W—H3WA	0.7423
C15—C16	1.518 (5)	O3W—H3WB	0.7034
C17—C18	1.392 (5)	O4W—H4WA	0.8913
C17—H17	0.9300	O4W—H4WB	0.7379
C18—C19	1.403 (5)	O5W—H5WA	0.77 (5)
C18—C22	1.513 (5)	O5W—H5WB	0.82 (5)
C19—C20	1.390 (5)	O6W—H6WA	0.7603
C19—C21	1.516 (5)	O6W—H6WB	0.8715
C20—H20	0.9300	O7W—H7WA	0.8486
C23—N9	1.329 (5)	O7W—H7WB	0.8493
C23—C24	1.395 (6)		
			
O3—Ni1—O1W	93.20 (11)	C25—C24—H24	120.6
O3—Ni1—N8	173.58 (11)	C23—C24—H24	120.6
O1W—Ni1—N8	91.69 (13)	C24—C25—C26	120.2 (4)
O3—Ni1—O2W	90.27 (11)	C24—C25—H25	119.9
O1W—Ni1—O2W	89.78 (11)	C26—C25—H25	119.9
N8—Ni1—O2W	93.92 (12)	C27—C26—C25	116.8 (4)
O3—Ni1—N7	96.05 (11)	C27—C26—C28	118.9 (4)
O1W—Ni1—N7	89.19 (12)	C25—C26—C28	124.3 (4)
N8—Ni1—N7	79.85 (13)	N9—C27—C26	123.3 (4)
O2W—Ni1—N7	173.65 (11)	N9—C27—C31	116.6 (3)
O3—Ni1—O2	86.80 (11)	C26—C27—C31	120.1 (4)
O1W—Ni1—O2	172.50 (10)	C29—C28—C26	121.1 (4)
N8—Ni1—O2	88.89 (13)	C29—C28—H28	119.5
O2W—Ni1—O2	82.73 (11)	C26—C28—H28	119.5
N7—Ni1—O2	98.27 (12)	C28—C29—C30	121.7 (4)
O6—Ni2—O4W	92.36 (11)	C28—C29—H29	119.2
O6—Ni2—N10	174.83 (11)	C30—C29—H29	119.2
O4W—Ni2—N10	91.26 (13)	C32—C30—C31	116.8 (4)
O6—Ni2—O3W	90.41 (11)	C32—C30—C29	124.6 (4)
O4W—Ni2—O3W	91.38 (11)	C31—C30—C29	118.6 (4)
N10—Ni2—O3W	93.20 (12)	N10—C31—C30	122.7 (4)
O6—Ni2—N9	96.53 (12)	N10—C31—C27	117.7 (3)
O4W—Ni2—N9	86.44 (12)	C30—C31—C27	119.5 (4)
N10—Ni2—N9	79.99 (13)	C33—C32—C30	120.5 (4)
O3W—Ni2—N9	172.79 (12)	C33—C32—H32	119.8
O6—Ni2—O7	87.05 (12)	C30—C32—H32	119.8
O4W—Ni2—O7	172.34 (11)	C32—C33—C34	119.4 (4)
N10—Ni2—O7	89.86 (13)	C32—C33—H33	120.3
O3W—Ni2—O7	80.99 (11)	C34—C33—H33	120.3
N9—Ni2—O7	101.22 (12)	N10—C34—C33	121.7 (5)
O11—Ni3—O9	89.64 (12)	N10—C34—H34	119.2
O11—Ni3—O5W	91.36 (12)	C33—C34—H34	119.2
O9—Ni3—O5W	85.65 (13)	N11—C35—C36	122.5 (5)
O11—Ni3—O6W	91.95 (11)	N11—C35—H35	118.8
O9—Ni3—O6W	170.55 (10)	C36—C35—H35	118.8
O5W—Ni3—O6W	85.00 (12)	C37—C36—C35	119.7 (4)
O11—Ni3—N11	174.19 (12)	C37—C36—H36	120.2
O9—Ni3—N11	89.31 (13)	C35—C36—H36	120.2
O5W—Ni3—N11	94.27 (13)	C36—C37—C38	119.8 (4)
O6W—Ni3—N11	90.02 (13)	C36—C37—H37	120.1
O11—Ni3—N12	94.73 (12)	C38—C37—H37	120.1
O9—Ni3—N12	95.94 (12)	C39—C38—C37	116.8 (4)
O5W—Ni3—N12	173.72 (12)	C39—C38—C40	119.5 (4)
O6W—Ni3—N12	93.21 (12)	C37—C38—C40	123.7 (4)
N11—Ni3—N12	79.70 (13)	N11—C39—C38	123.5 (4)
C13—O2—Ni1	122.2 (3)	N11—C39—C43	117.4 (3)
C16—O3—Ni1	121.0 (2)	C38—C39—C43	119.2 (4)
C21—O6—Ni2	119.8 (2)	C41—C40—C38	121.6 (4)
C22—O7—Ni2	120.7 (3)	C41—C40—H40	119.2
C47—O9—Ni3	139.8 (2)	C38—C40—H40	119.2
C51—O11—Ni3	128.3 (2)	C40—C41—C42	120.8 (4)
N7—C1—C2	122.6 (4)	C40—C41—H41	119.6
N7—C1—H1	118.7	C42—C41—H41	119.6
C2—C1—H1	118.7	C44—C42—C43	117.1 (4)
C3—C2—C1	118.9 (4)	C44—C42—C41	124.4 (4)
C3—C2—H2	120.5	C43—C42—C41	118.5 (4)
C1—C2—H2	120.5	N12—C43—C42	122.7 (4)
C2—C3—C4	120.4 (4)	N12—C43—C39	116.7 (3)
C2—C3—H3	119.8	C42—C43—C39	120.5 (4)
C4—C3—H3	119.8	C45—C44—C42	120.0 (4)
C3—C4—C5	116.9 (4)	C45—C44—H44	120.0
C3—C4—C6	124.8 (4)	C42—C44—H44	120.0
C5—C4—C6	118.3 (4)	C44—C45—C46	119.0 (4)
N7—C5—C4	122.7 (4)	C44—C45—H45	120.5
N7—C5—C9	117.0 (3)	C46—C45—H45	120.5
C4—C5—C9	120.2 (4)	N12—C46—C45	123.1 (4)
C7—C6—C4	121.9 (4)	N12—C46—H46	118.5
C7—C6—H6	119.0	C45—C46—H46	118.5
C4—C6—H6	119.0	O10—C47—O9	124.5 (3)
C6—C7—C8	121.0 (4)	O10—C47—C48	117.5 (3)
C6—C7—H7	119.5	O9—C47—C48	118.0 (3)
C8—C7—H7	119.5	C50—C48—C49	118.3 (3)
C10—C8—C9	117.2 (4)	C50—C48—C47	123.9 (3)
C10—C8—C7	123.9 (4)	C49—C48—C47	117.7 (3)
C9—C8—C7	118.9 (4)	C50^i^—C49—C48	122.1 (3)
N8—C9—C8	123.0 (4)	C50^i^—C49—H49	118.9
N8—C9—C5	117.4 (3)	C48—C49—H49	118.9
C8—C9—C5	119.6 (4)	C49^i^—C50—C48	119.6 (3)
C11—C10—C8	119.3 (4)	C49^i^—C50—C51	116.4 (3)
C11—C10—H10	120.3	C48—C50—C51	124.1 (3)
C8—C10—H10	120.3	O12—C51—O11	123.3 (3)
C10—C11—C12	120.4 (4)	O12—C51—C50	116.5 (3)
C10—C11—H11	119.8	O11—C51—C50	120.0 (3)
C12—C11—H11	119.8	C1—N7—C5	118.4 (3)
N8—C12—C11	121.8 (4)	C1—N7—Ni1	129.1 (3)
N8—C12—H12	119.1	C5—N7—Ni1	112.5 (2)
C11—C12—H12	119.1	C12—N8—C9	118.2 (4)
O1—C13—O2	124.4 (3)	C12—N8—Ni1	128.8 (3)
O1—C13—C14	116.7 (3)	C9—N8—Ni1	113.0 (2)
O2—C13—C14	118.9 (3)	C23—N9—C27	117.6 (3)
C20—C14—C15	119.3 (3)	C23—N9—Ni2	129.4 (3)
C20—C14—C13	117.9 (3)	C27—N9—Ni2	113.0 (2)
C15—C14—C13	122.9 (3)	C34—N10—C31	118.9 (4)
C17—C15—C14	118.1 (3)	C34—N10—Ni2	128.4 (3)
C17—C15—C16	116.3 (3)	C31—N10—Ni2	112.7 (3)
C14—C15—C16	125.6 (3)	C35—N11—C39	117.8 (4)
O4—C16—O3	124.4 (3)	C35—N11—Ni3	129.3 (3)
O4—C16—C15	118.2 (3)	C39—N11—Ni3	112.9 (2)
O3—C16—C15	116.9 (3)	C46—N12—C43	117.9 (3)
C15—C17—C18	122.8 (3)	C46—N12—Ni3	129.5 (3)
C15—C17—H17	118.6	C43—N12—Ni3	112.6 (2)
C18—C17—H17	118.6	Ni1—O1W—H1WA	119.8
C17—C18—C19	119.3 (3)	Ni1—O1W—H1WB	103.6
C17—C18—C22	117.9 (3)	H1WA—O1W—H1WB	108.7
C19—C18—C22	122.8 (3)	Ni1—O2W—H2WA	113.8
C20—C19—C18	118.1 (3)	Ni1—O2W—H2WB	117.3
C20—C19—C21	115.5 (3)	H2WA—O2W—H2WB	113.6
C18—C19—C21	126.2 (3)	Ni2—O3W—H3WA	114.0
C19—C20—C14	122.4 (3)	Ni2—O3W—H3WB	111.8
C19—C20—H20	118.8	H3WA—O3W—H3WB	119.0
C14—C20—H20	118.8	Ni2—O4W—H4WA	110.3
O5—C21—O6	124.5 (3)	Ni2—O4W—H4WB	122.7
O5—C21—C19	119.5 (3)	H4WA—O4W—H4WB	102.0
O6—C21—C19	115.5 (3)	Ni3—O5W—H5WA	110 (4)
O8—C22—O7	124.5 (3)	Ni3—O5W—H5WB	120 (3)
O8—C22—C18	118.4 (3)	H5WA—O5W—H5WB	104 (5)
O7—C22—C18	117.1 (3)	Ni3—O6W—H6WA	123.6
N9—C23—C24	123.1 (4)	Ni3—O6W—H6WB	110.5
N9—C23—H23	118.4	H6WA—O6W—H6WB	100.2
C24—C23—H23	118.4	H7WA—O7W—H7WB	107.9
C25—C24—C23	118.8 (4)		
			
O3—Ni1—O2—C13	−85.8 (3)	C38—C40—C41—C42	1.0 (8)
O1W—Ni1—O2—C13	−176.1 (7)	C40—C41—C42—C44	−179.9 (5)
N8—Ni1—O2—C13	89.4 (3)	C40—C41—C42—C43	−0.7 (7)
O2W—Ni1—O2—C13	−176.5 (3)	C44—C42—C43—N12	1.7 (6)
N7—Ni1—O2—C13	9.8 (3)	C41—C42—C43—N12	−177.5 (4)
O1W—Ni1—O3—C16	−172.7 (3)	C44—C42—C43—C39	179.6 (4)
N8—Ni1—O3—C16	−33.2 (13)	C41—C42—C43—C39	0.4 (6)
O2W—Ni1—O3—C16	97.5 (3)	N11—C39—C43—N12	−1.3 (6)
N7—Ni1—O3—C16	−83.2 (3)	C38—C39—C43—N12	177.7 (4)
O2—Ni1—O3—C16	14.8 (3)	N11—C39—C43—C42	−179.3 (4)
O4W—Ni2—O6—C21	173.0 (3)	C38—C39—C43—C42	−0.3 (6)
N10—Ni2—O6—C21	38.8 (16)	C43—C42—C44—C45	−2.8 (7)
O3W—Ni2—O6—C21	−95.6 (3)	C41—C42—C44—C45	176.3 (5)
N9—Ni2—O6—C21	86.4 (3)	C42—C44—C45—C46	0.1 (8)
O7—Ni2—O6—C21	−14.6 (3)	C44—C45—C46—N12	4.2 (7)
O6—Ni2—O7—C22	89.0 (3)	Ni3—O9—C47—O10	−177.5 (3)
O4W—Ni2—O7—C22	174.8 (7)	Ni3—O9—C47—C48	0.5 (6)
N10—Ni2—O7—C22	−86.8 (3)	O10—C47—C48—C50	−135.4 (4)
O3W—Ni2—O7—C22	179.9 (3)	O9—C47—C48—C50	46.4 (5)
N9—Ni2—O7—C22	−7.0 (3)	O10—C47—C48—C49	42.8 (5)
O11—Ni3—O9—C47	−47.2 (4)	O9—C47—C48—C49	−135.4 (4)
O5W—Ni3—O9—C47	−138.6 (4)	C50—C48—C49—C50^i^	0.1 (6)
O6W—Ni3—O9—C47	−147.0 (6)	C47—C48—C49—C50^i^	−178.2 (4)
N11—Ni3—O9—C47	127.1 (4)	C49—C48—C50—C49^i^	−0.1 (6)
N12—Ni3—O9—C47	47.5 (4)	C47—C48—C50—C49^i^	178.1 (4)
O9—Ni3—O11—C51	16.6 (3)	C49—C48—C50—C51	−177.9 (4)
O5W—Ni3—O11—C51	102.2 (3)	C47—C48—C50—C51	0.3 (6)
O6W—Ni3—O11—C51	−172.7 (3)	Ni3—O11—C51—O12	−137.6 (3)
N11—Ni3—O11—C51	−63.0 (14)	Ni3—O11—C51—C50	47.6 (5)
N12—Ni3—O11—C51	−79.3 (3)	C49^i^—C50—C51—O12	−66.9 (5)
N7—C1—C2—C3	2.9 (7)	C48—C50—C51—O12	111.0 (4)
C1—C2—C3—C4	0.2 (7)	C49^i^—C50—C51—O11	108.2 (4)
C2—C3—C4—C5	−2.3 (6)	C48—C50—C51—O11	−73.9 (5)
C2—C3—C4—C6	175.8 (4)	C2—C1—N7—C5	−3.7 (6)
C3—C4—C5—N7	1.6 (6)	C2—C1—N7—Ni1	173.8 (3)
C6—C4—C5—N7	−176.6 (4)	C4—C5—N7—C1	1.3 (6)
C3—C4—C5—C9	−180.0 (4)	C9—C5—N7—C1	−177.1 (3)
C6—C4—C5—C9	1.8 (6)	C4—C5—N7—Ni1	−176.5 (3)
C3—C4—C6—C7	−179.2 (4)	C9—C5—N7—Ni1	5.0 (4)
C5—C4—C6—C7	−1.1 (7)	O3—Ni1—N7—C1	−7.0 (3)
C4—C6—C7—C8	0.5 (7)	O1W—Ni1—N7—C1	86.2 (3)
C6—C7—C8—C10	178.4 (4)	N8—Ni1—N7—C1	178.0 (4)
C6—C7—C8—C9	−0.6 (7)	O2W—Ni1—N7—C1	166.8 (9)
C10—C8—C9—N8	1.3 (6)	O2—Ni1—N7—C1	−94.6 (3)
C7—C8—C9—N8	−179.7 (4)	O3—Ni1—N7—C5	170.6 (3)
C10—C8—C9—C5	−177.8 (4)	O1W—Ni1—N7—C5	−96.3 (3)
C7—C8—C9—C5	1.2 (6)	N8—Ni1—N7—C5	−4.4 (3)
N7—C5—C9—N8	−2.5 (5)	O2W—Ni1—N7—C5	−15.6 (12)
C4—C5—C9—N8	179.0 (4)	O2—Ni1—N7—C5	83.0 (3)
N7—C5—C9—C8	176.6 (3)	C11—C12—N8—C9	−2.0 (7)
C4—C5—C9—C8	−1.9 (6)	C11—C12—N8—Ni1	179.0 (4)
C9—C8—C10—C11	−1.2 (7)	C8—C9—N8—C12	0.3 (6)
C7—C8—C10—C11	179.8 (5)	C5—C9—N8—C12	179.4 (4)
C8—C10—C11—C12	−0.3 (8)	C8—C9—N8—Ni1	179.5 (3)
C10—C11—C12—N8	2.0 (9)	C5—C9—N8—Ni1	−1.5 (4)
Ni1—O2—C13—O1	−128.4 (4)	O3—Ni1—N8—C12	131.4 (11)
Ni1—O2—C13—C14	52.9 (4)	O1W—Ni1—N8—C12	−89.0 (4)
O1—C13—C14—C20	21.5 (5)	O2W—Ni1—N8—C12	0.9 (4)
O2—C13—C14—C20	−159.7 (4)	N7—Ni1—N8—C12	−177.8 (4)
O1—C13—C14—C15	−159.6 (4)	O2—Ni1—N8—C12	83.6 (4)
O2—C13—C14—C15	19.2 (6)	O3—Ni1—N8—C9	−47.6 (13)
C20—C14—C15—C17	−1.1 (6)	O1W—Ni1—N8—C9	92.0 (3)
C13—C14—C15—C17	179.9 (3)	O2W—Ni1—N8—C9	−178.1 (3)
C20—C14—C15—C16	175.2 (4)	N7—Ni1—N8—C9	3.1 (3)
C13—C14—C15—C16	−3.7 (6)	O2—Ni1—N8—C9	−95.5 (3)
Ni1—O3—C16—O4	−128.9 (3)	C24—C23—N9—C27	5.0 (6)
Ni1—O3—C16—C15	59.4 (4)	C24—C23—N9—Ni2	−177.0 (3)
C17—C15—C16—O4	−71.3 (5)	C26—C27—N9—C23	−3.2 (6)
C14—C15—C16—O4	112.3 (4)	C31—C27—N9—C23	176.3 (4)
C17—C15—C16—O3	101.0 (4)	C26—C27—N9—Ni2	178.4 (3)
C14—C15—C16—O3	−75.4 (5)	C31—C27—N9—Ni2	−2.1 (4)
C14—C15—C17—C18	−0.2 (6)	O6—Ni2—N9—C23	6.9 (4)
C16—C15—C17—C18	−176.8 (4)	O4W—Ni2—N9—C23	−85.0 (4)
C15—C17—C18—C19	1.7 (6)	N10—Ni2—N9—C23	−176.9 (4)
C15—C17—C18—C22	−178.6 (4)	O3W—Ni2—N9—C23	−157.5 (8)
C17—C18—C19—C20	−1.9 (6)	O7—Ni2—N9—C23	95.2 (4)
C22—C18—C19—C20	178.4 (3)	O6—Ni2—N9—C27	−174.9 (3)
C17—C18—C19—C21	−177.2 (4)	O4W—Ni2—N9—C27	93.1 (3)
C22—C18—C19—C21	3.1 (6)	N10—Ni2—N9—C27	1.2 (3)
C18—C19—C20—C14	0.7 (6)	O3W—Ni2—N9—C27	20.6 (11)
C21—C19—C20—C14	176.5 (4)	O7—Ni2—N9—C27	−86.6 (3)
C15—C14—C20—C19	0.8 (6)	C33—C34—N10—C31	1.1 (7)
C13—C14—C20—C19	179.9 (4)	C33—C34—N10—Ni2	−178.4 (4)
Ni2—O6—C21—O5	127.8 (4)	C30—C31—N10—C34	−0.9 (6)
Ni2—O6—C21—C19	−60.2 (4)	C27—C31—N10—C34	179.4 (4)
C20—C19—C21—O5	73.9 (5)	C30—C31—N10—Ni2	178.7 (3)
C18—C19—C21—O5	−110.7 (5)	C27—C31—N10—Ni2	−1.0 (5)
C20—C19—C21—O6	−98.5 (4)	O6—Ni2—N10—C34	−132.4 (14)
C18—C19—C21—O6	76.9 (5)	O4W—Ni2—N10—C34	93.3 (4)
Ni2—O7—C22—O8	126.0 (4)	O3W—Ni2—N10—C34	1.9 (4)
Ni2—O7—C22—C18	−55.6 (4)	N9—Ni2—N10—C34	179.5 (4)
C17—C18—C22—O8	−18.7 (6)	O7—Ni2—N10—C34	−79.1 (4)
C19—C18—C22—O8	161.0 (4)	O6—Ni2—N10—C31	48.1 (16)
C17—C18—C22—O7	162.9 (4)	O4W—Ni2—N10—C31	−86.3 (3)
C19—C18—C22—O7	−17.4 (6)	O3W—Ni2—N10—C31	−177.7 (3)
N9—C23—C24—C25	−2.9 (7)	N9—Ni2—N10—C31	−0.1 (3)
C23—C24—C25—C26	−1.2 (7)	O7—Ni2—N10—C31	101.3 (3)
C24—C25—C26—C27	2.7 (7)	C36—C35—N11—C39	1.9 (7)
C24—C25—C26—C28	−176.4 (4)	C36—C35—N11—Ni3	−175.3 (4)
C25—C26—C27—N9	−0.5 (6)	C38—C39—N11—C35	−2.0 (6)
C28—C26—C27—N9	178.7 (4)	C43—C39—N11—C35	177.0 (4)
C25—C26—C27—C31	180.0 (4)	C38—C39—N11—Ni3	175.7 (3)
C28—C26—C27—C31	−0.8 (6)	C43—C39—N11—Ni3	−5.3 (5)
C27—C26—C28—C29	−0.7 (7)	O11—Ni3—N11—C35	167.8 (11)
C25—C26—C28—C29	178.4 (5)	O9—Ni3—N11—C35	88.1 (4)
C26—C28—C29—C30	1.1 (7)	O5W—Ni3—N11—C35	2.5 (4)
C28—C29—C30—C32	179.2 (5)	O6W—Ni3—N11—C35	−82.5 (4)
C28—C29—C30—C31	0.0 (7)	N12—Ni3—N11—C35	−175.7 (4)
C32—C30—C31—N10	−0.5 (6)	O11—Ni3—N11—C39	−9.6 (15)
C29—C30—C31—N10	178.8 (4)	O9—Ni3—N11—C39	−89.3 (3)
C32—C30—C31—C27	179.2 (4)	O5W—Ni3—N11—C39	−174.8 (3)
C29—C30—C31—C27	−1.5 (6)	O6W—Ni3—N11—C39	100.2 (3)
N9—C27—C31—N10	2.2 (5)	N12—Ni3—N11—C39	6.9 (3)
C26—C27—C31—N10	−178.3 (4)	C45—C46—N12—C43	−5.2 (6)
N9—C27—C31—C30	−177.6 (4)	C45—C46—N12—Ni3	171.3 (3)
C26—C27—C31—C30	1.9 (6)	C42—C43—N12—C46	2.2 (6)
C31—C30—C32—C33	1.7 (7)	C39—C43—N12—C46	−175.7 (4)
C29—C30—C32—C33	−177.5 (5)	C42—C43—N12—Ni3	−174.9 (3)
C30—C32—C33—C34	−1.5 (8)	C39—C43—N12—Ni3	7.2 (4)
C32—C33—C34—N10	0.1 (8)	O11—Ni3—N12—C46	−5.9 (4)
N11—C35—C36—C37	−0.6 (8)	O9—Ni3—N12—C46	−96.1 (4)
C35—C36—C37—C38	−0.8 (8)	O5W—Ni3—N12—C46	159.5 (11)
C36—C37—C38—C39	0.8 (7)	O6W—Ni3—N12—C46	86.3 (4)
C36—C37—C38—C40	−178.0 (5)	N11—Ni3—N12—C46	175.7 (4)
C37—C38—C39—N11	0.6 (7)	O11—Ni3—N12—C43	170.7 (3)
C40—C38—C39—N11	179.5 (4)	O9—Ni3—N12—C43	80.6 (3)
C37—C38—C39—C43	−178.4 (4)	O5W—Ni3—N12—C43	−23.8 (13)
C40—C38—C39—C43	0.5 (6)	O6W—Ni3—N12—C43	−97.0 (3)
C39—C38—C40—C41	−0.9 (7)	N11—Ni3—N12—C43	−7.6 (3)
C37—C38—C40—C41	177.9 (5)		

Symmetry code: (i) −*x*, −*y*, −*z*+1.

### Hydrogen-bond geometry (Å, º)

**Table:**

*D*—H···*A*	*D*—H	H···*A*	*D*···*A*	*D*—H···*A*
O1*W*—H1*WA*···O4^ii^	0.81	1.97	2.774 (4)	176
O1*W*—H1*WB*···O8^iii^	0.88	1.81	2.667 (4)	163
O2*W*—H2*WA*···O7^iv^	0.82	2.32	3.122 (4)	168
O2*W*—H2*WB*···O3^ii^	0.77	2.00	2.740 (4)	161
O3*W*—H3*WA*···O11	0.74	2.05	2.767 (4)	163
O3*W*—H3*WB*···O2^v^	0.70	2.44	3.120 (4)	164
O4*W*—H4*WA*···O10^vi^	0.89	1.87	2.746 (4)	166
O5*W*—H5*WA*···O6	0.78 (4)	1.92 (4)	2.692 (4)	173 (4)
O6*W*—H6*WA*···O5	0.76	2.12	2.874 (4)	174
O6*W*—H6*WB*···O1^v^	0.87	1.80	2.622 (4)	156
O7*W*—H7*WA*···O9^vii^	0.85	2.05	2.893 (6)	172
O7*W*—H7*WB*···O12^viii^	0.85	2.15	2.797 (6)	132

Symmetry codes: (ii) −*x*+3, −*y*, −*z*; (iii) −*x*+2, −*y*, −*z*; (iv) *x*+1, *y*, *z*; (v) *x*−1, *y*, *z*; (vi) −*x*+1, −*y*, −*z*+1; (vii) −*x*+1, −*y*+1, −*z*+1; (viii) *x*, *y*+1, *z*.
